# A Longitudinal Evaluation of the Effects of Moderate Physical Activity on Health, Metabolic, and Physiological Parameters in Individuals With Type 2 Diabetes Mellitus: A Comprehensive Study

**DOI:** 10.7759/cureus.67370

**Published:** 2024-08-21

**Authors:** Ruchi Kothari, Abhishek Burra, Rajiv Ranjan, Deepak C Rathod, Mohit Agrawal, Tejaswee Lohakare, Prashanth A

**Affiliations:** 1 Physiology, Mahatma Gandhi Institute of Medical Sciences, Wardha, IND; 2 Emergency Medicine, Aadhya Hospital, Khammam, IND; 3 Physiology, Jawaharlal Nehru Medical College, Aligarh Muslim University, Aligarh, IND; 4 General Medicine, Welfare Hospital, Bhatkal, IND; 5 Physical Medicine and Rehabilitation, S. S. Agrawal Institute of Physiotherapy and Medical Care Education, Navsari, IND; 6 Child Health Nursing, Srimati Radhikabai Meghe Memorial College of Nursing, Datta Meghe Institute of Higher Education and Research, Wardha, IND

**Keywords:** developing nation, activity level, t2dm, hyperglycemia, physical training

## Abstract

Background

Diabetes mellitus (DM), especially type 2 diabetes mellitus (T2DM), is a serious worldwide health concern that is becoming more common and often develops at a young age, particularly in developing countries. Given the increasing incidence of diabetes in India, efficient management techniques are advantageous. The aim of this study is to evaluate the impact of physical activity on body measurements, biochemical markers, and clinical outcomes in Indian individuals with T2DM.

Methodology

Utilizing a universal sampling approach, a longitudinal interventional study was performed with T2DM patients at a metropolitan health and training facility over an eight-month duration. Over the course of six months, they were told to walk briskly for 2.5 hours each week. Initial and follow-up evaluations included monitoring arterial pressure, body mass, abdominal circumference, and glucose concentrations. During monthly follow-ups, participants self-reported their compliance.

Results

The study of 210 participants shows a diverse age distribution with the majority in the 35-54 years range and a higher number of females (56.19%) compared to males (43.81%). Most were Hindu (77.6%), with varying education levels and a predominance of unemployment (69.5%) and marital status (91.0% married). Post-exercise, significant improvements were observed in fasting blood glucose (160.45 to 140.20 mg/dL; p = 0.004), postprandial blood glucose (270.35 to 240.55 mg/dL; p = 0.002), body weight (70.00 to 66.50 kg; p = 0.03), and waist circumference (95.00 to 90.50 cm; p = 0.01).

Conclusion

The outcomes of this prolonged study reveal that along with promoting weight reduction in T2DM patients, regular engagement in moderate exercise can substantially improve both fasting and post-meal blood glucose levels.

## Introduction

Type 1 and type 2 diabetes mellitus (T2DM) are lifestyle-related diseases characterized by high blood sugar levels due to an absolute or relative deficiency of the hormone insulin [1]. An estimated 350 million individuals worldwide are believed to have diabetes, and if nothing is done, this number is predicted to quadruple by 2030 [2]. Despite the long-held belief that diabetes is more prevalent in industrialized countries, new data indicates that emerging countries have experienced a notable increase in diabetes incidence and earlier age of start [3]. Furthermore, diabetes is predicted to rank seventh in terms of causes of mortality by 2030 and accounts for about 80% of fatalities in low- and middle-income countries [4].

India has earned the nickname "diabetes capital of the world" because it has the highest proportion of individuals with diabetes [5]. In fact, compared to 32 million in 2000, it is predicted that there would be about 80 million cases of diabetes by 2030 [5]. The causes of diabetes mellitus (DM) are complex and include both modifiable (such as obesity, sedentary lifestyle, nutrition, and genetic flaws) and nonmodifiable (such as age, family history, and genetic defects) factors [6]. Since the beginning of time, people have understood the importance of exercise in the prevention and management of diabetes [7]. Exercise and physical activity of some kind have really been recommended for the early and continuous control of T2DM [8].

Exercise has been linked to a multitude of benefits for diabetics, including better glycemic control, decreased blood pressure, reduced low-density lipoproteins (LDL) levels, improved weight reduction, sustained weight management, balance during walking, quicker response time, better posture, and preservation of autonomic balance increased insulin receptor sensitivity and number, and decreased glycated hemoglobin (HbA1c) [9]. Moreover, a greater percentage of people with diabetes may be able to reduce or even stop using their medications thanks to increased physical activity [10].

Considering the importance of physical exercise in managing T2DM and the scarcity of research on its impact on different aspects of the disease, especially in the Indian population, this study seeks to assess the level of physical activity among T2DM patients.

## Materials and methods

Study design and setting

Over the course of eight months, a longitudinal interventional study was performed with T2DM patients at a metropolitan health and training facility. Patients diagnosed with T2DM were recruited in the trial as long as they fit the inclusion and exclusion criteria, thanks to the universal sampling technique that was used. The institutional ethics committee gave its permission before the study could start.

Selection Criteria

In order to be eligible for the study, a patient had to be a type 2 diabetic, have normal electrocardiogram (ECG) results prior to enrolment, demonstrate a typical blood pressure reaction to standing (a decrease in systolic pressure to below 20 mmHg and diastolic pressure to under 10 mmHg), have no major peripheral vascular disease, and exhibit no signs of retinopathy or macular edema upon fundoscopy. On the other hand, individuals who were not willing to take part in the study or who intended to move within the following six months, or who were receiving regular short-acting human insulin treatment, or concomitant illnesses such as bronchial asthma or advanced hypertension with associated organ damage, were excluded from the study.

Data sources and variables

The research participants' sociodemographic and economic information was gathered using a semi-structured questionnaire. Following selection, these subjects underwent blood pressure, weight, and waist circumference measurements as well as estimations of postprandial and fasting blood sugar. The outcomes that were acquired served as the foundational information.

The benefits of physical activity and exercise were explained to each selected diabetic patient. Participants were advised to engage in brisk walking for 2.5 hours per week, equivalent to 30 minutes per day, five days a week, as a moderate form of cardiovascular exercise [11]. Counseling was provided on recognizing hypoglycemia symptoms, such as giddiness, excessive sweating, or uneasiness, and patients were advised to carry glucose biscuits during walks. They were instructed to consume biscuits if symptoms occurred during exercise, report any discomfort to the hospital, and maintain regular monthly follow-ups.

To monitor exercise adherence, study participants were asked to self-report their compliance with the prescribed exercise routine during their monthly follow-up visits. Additionally, participants were provided with activity logs to record their daily exercise duration and intensity. These logs were reviewed during follow-up appointments to verify adherence. Clinical evaluations were conducted at each follow-up, and patients were encouraged to continue the recommended physical activity levels. After six months, a final clinical evaluation, including arterial pressure, body mass, abdominal circumference, and glucose concentrations, was conducted. The results were compared to the baseline measurements.

Statistical analysis

IBM SPSS Statistics for Windows, Version 21 (Released 2012; IBM Corp., Armonk, New York, United States) was used for data entry and statistical analysis. For every variable, percentages and frequency distributions were calculated. Set to p < 0.05, the statistical significance was maintained. For continuous (mean ± SD) variables, paired t-tests were used to examine the relationships.

## Results

Table 1 describes the sociodemographic and economic characteristics of the study participants (n = 210). The age distribution is as follows: 25-34 years (28 participants; 13.3%), 35-44 years (56 participants; 26.7%), 45-54 years (62 participants; 29.5%), 55-64 years (42 participants; 20.0%), and 65-74 years (22 participants; 10.5%). Regarding gender, there are 92 males (43.81%) and 118 females (56.19%). In terms of religious affiliation, 163 participants (77.6%) are Hindu, while 47 participants (22.4%) are either Muslim or Christian. The educational level of participants is categorized as follows: no formal education (38 participants, 18.1%), primary education (110 participants, 52.4%), and secondary education or higher (62 participants, 29.5%). Employment status shows that 64 participants (30.5%) are employed, and 146 participants (69.5%) are unemployed. Marital status reveals that 191 participants (91.0%) are married, while 19 participants (9.0%) are single.

**Table 1:** Demographic and socioeconomic attributes of study participants (n = 210)

Attribute	Classification	Number (%)
Age range (years)	25-34	28 (13.3)
	35-44	56 (26.7)
	45-54	62 (29.5)
	55-64	42 (20.0)
	65-74	22 (10.5)
Sex	Men	92 (43.81)
	Women	118 (56.19)
Religious affiliation	Hindu	163 (77.6)
	Muslim/Christian	47 (22.4)
Level of education	No formal schooling	3 818.1)
	Basic education (primary education)	110 (52.4)
	Secondary education and above	62 (29.5)
Employment status	Employed	64 (30.5)
	Unemployed	146 (69.5)
Marital status	Married	191 (91.0)
	Single	19 (9.0)

Table 2 provides a comparative analysis of anthropometric and biochemical parameters pre- and post-exercise (n = 210). The mean fasting blood glucose level before exercise is 160.45 ± 70.50 mg/dL, which decreases to 140.20 ± 58.30 mg/dL after exercise, with a p-value of 0.004. The mean postprandial blood glucose level before exercise is 270.35 ± 110.20 mg/dL, which decreases to 240.55 ± 100.45 mg/dL after exercise, with a p-value of 0.002. The mean body weight of participants before exercise is 70.00 ± 15.00 kg, which reduces to 66.50 ± 14.00 kg after exercise, with a p-value of 0.03. The mean waist measurement before exercise is 95.00 ± 9.00 cm, which decreases to 90.50 ± 10.50 cm after exercise, with a p-value of 0.01. The mean body mass index (BMI) before exercise is 27.3 ± 4.5 kg/m^2^, which decreases to 25.8 ± 4.2 kg/m^2^ after exercise, with a p-value of 0.02.

**Table 2:** Comparative analysis of anthropometric and biochemical parameters pre- and post-exercise (n = 210) p-value < 0.05 is considered to be statistically significant

Attribute	Before exercise	After exercise	p-value
Fasting blood glucose (FBS)	160.45 ± 70.50	140.20 ± 58.30	0.004
Postprandial blood glucose (PPBS)	270.35 ± 110.20	240.55 ± 100.45	0.002
Body weight (kg)	70.00 ± 15.00	66.50 ± 14.00	0.03
Waist measurement (cm)	95.00 ± 9.00	90.50 ± 10.50	0.01
Body mass index (BMI)	27.3 ± 4.5	25.8 ± 4.2	0.02

## Discussion

This research offers a thorough examination of the effects of moderate exercise on individuals with T2DM, taking into account biochemical, anthropometric, and sociodemographic factors. According to the sociodemographic profile, a sizable fraction of participants was between the ages of 45 and 54. Females made up a larger percentage of the sample (56.19%) than did men (43.81%). This distribution of demographic characteristics is in line with other research findings that emphasize the prevalence of older individuals and females in T2DM communities [12,13]. The majority of individuals reflected common trends seen in diabetes research: they were married, of Hindu descent, with just a primary education and no job.

The study discovered substantial improvements in clinical outcomes after engaging in moderate exercise. The blood glucose levels after a meal reduced from 270.35 ± 110.20 mg/dL to 240.55 ± 100.45 mg/dL (p = 0.002), whereas the blood glucose levels during fasting decreased from 160.45 ± 70.50 mg/dL to 140.20 ± 58.30 mg/dL (p = 0.004). These reductions align with previous research demonstrating the positive effects of physical activity on blood sugar control [9,14,15]. Furthermore, waist circumference dropped from 95.00 ± 9.00 cm to 90.50 ± 10.50 cm (p = 0.01), and body weight dropped from 70.00 ± 15.00 kg to 66.50 ± 14.00 kg (p = 0.03), demonstrating the positive effects of physical exercise on body composition.

In addition to waist circumference and body weight, BMI was also evaluated, with a reduction observed from 27.3 ± 4.5 kg/m² to 25.8 ± 4.2 kg/m² (p = 0.02). This decrease in BMI further underscores the beneficial impact of moderate exercise on body composition. Maintaining a healthy BMI is crucial for T2DM patients as it is associated with better glycemic control, reduced insulin resistance, and lower risks of associated complications.

The metabolic mechanisms that physical activity triggers can be responsible for the advantages of exercise that have been shown. Exercise initially triggers the breakdown of muscle glycogen, subsequently leading to the utilization of free fatty acids and stored fats in adipose tissue to promote metabolic health [16,17]. Even though the weight loss was noteworthy, it was not as substantial as some other research that revealed more pronounced weight loss with comparable therapies [18,19]. This disparity might be connected to variations in exercise adherence or intensity among participants.

Beyond the immediate improvements, the potential long-term impacts of moderate physical activity should be considered. For instance, regular moderate exercise has been shown to significantly lower HbA1c levels, a crucial marker of long-term blood glucose control. Sustained reductions in HbA1c are associated with decreased risks of microvascular and macrovascular complications in T2DM patients. Additionally, moderate exercise can improve cardiovascular health by reducing blood pressure, improving lipid profiles, and enhancing endothelial function, thereby lowering the risk of cardiovascular events, which are common in T2DM populations [12,16].

Furthermore, the current study's findings closely resemble those of Sukla et al., who also noted significant improvements in blood glucose levels and body composition following a regimen of moderate physical activity [20]. This similarity reinforces the notion that moderate exercise can be an effective intervention for managing T2DM. However, the variations in weight loss outcomes between studies highlight the importance of individualized exercise programs and the need for further research to optimize exercise recommendations for T2DM patients.

In addition to the biochemical and anthropometric improvements, the study's focus on the sociodemographic aspects of T2DM patients provides valuable insights. The higher prevalence of females and older adults with T2DM suggests targeted public health interventions are necessary to address these vulnerable groups. The educational and occupational status of participants also underscores the need for accessible exercise programs that can be easily integrated into the daily routines of individuals with varying educational backgrounds and employment statuses.

Overall, this research contributes to the growing body of evidence supporting the benefits of moderate exercise for individuals with T2DM. The findings highlight the multifaceted impact of physical activity, encompassing improvements in blood glucose control, body composition, and metabolic health, while also addressing important sociodemographic factors. Future studies should continue to explore the long-term effects of different types and intensities of exercise, as well as strategies to enhance adherence and optimize outcomes for diverse populations with T2DM.

Limitations of the study

The lack of a control group and exercise adherence monitoring are limitations that might limit how broadly the results can be applied. To further assess the usefulness of exercise in managing diabetes, future studies should overcome these shortcomings and include new metrics, such as HbA1c values.

## Conclusions

The results of this long-term trial indicate that in addition to helping T2DM patients lose weight, compliance with modest physical exercise can greatly improve fasting and postprandial blood sugar levels. Additionally, the study's findings may be used by program administrators to emphasize physical exercise as a critical element in making sure diabetes patients' blood sugar is well controlled. Therefore, physical exercise guarantees a comprehensive strategy for effective control of diabetes mellitus when combined with therapeutic management of the disease.
